# Dr. Kinglake, in Reply to Mr. Ring, on the Origin of Cow-Pock Matter

**Published:** 1806-10

**Authors:** Robert Kinglake

**Affiliations:** Taunton


### Dr. Kinglake, in Reply to Mr. Ring, on the Origin of Cow-Pock Matter.

*To the Editors of the Medical and Physical Journal.*

GENTLEMEN,

MR. RING'S answer to my paper, respecting the source of the fluid which produces the genuine cow-pock disease, was not wanting to prove his talent at asperity and satire. Amidst much ingenious and acute investigation of the merits of vaccine inoculation, it must be impartially confessed that Mr. Ring has suffered himself to be betrayed into much unguarded and undue severity of censure. He seems to regard every insinuation against the validity of his favourite inquiry, as taking an unpardonable liberty with his opinion of its incontrovertible clearness.

Mr. Ring perceives, in his own persuasion respecting the nature and effects of the vaccine disease, a *non tangendum* something, a demonstrative certainty, that would imply equal "ignorance" and illiberality for a moment to question. This attempt to force argument by violence, confers no legitimate authority, and but rarely succeeds in obtaining rational converts.

The intrinsic merits of vaccine inoculation are of a nature to command a patient hearing, and need not, by any means, the enthusiastic vehemence of impassioned zeal. Mr. Ring may write volumes, in addition to what he has already offered to the public, but will not advance the true interests of vaccine inoculation but in the exact proportion in which he candidly and dispassionately discusses every occurring circumstance, involving any new question relative to the practice. Mr. Ring considers the arduous inquiry at an end, as having reached the happy issue expected, and that the subject is now become "hackneyed and threadbare." This may be the case in his own private estimation, but it is probably not so either in the medical or popular sentiment of mankind.

Vaccine inoculation has my implicit confidence; my infant children have been subjected to its benign influence, which, it is presumed, may be justly admitted as a sure and solemn pledge of my fidelity. With this persuasion, it must be allowed, that my solicitude as to the source of the disease, is neither capricious nor unimportant; it naturally arises from an ardent wish to see the hopeful hopeful practice fenced round by a mass of evidence that shall present to the unprejudiced, a clear and comprehensive knowledge of the origin, nature, and effects of the distemper. It will still become me therefore, as well as every person feeling the momentous nature of the subject, to endeavour to ascertain the genuine source of the disease by renewed and varied appeals to actual experiment.

Mr. Ring begs to have pointed out to him, where he has been so "unguarded" as to say, that the practice of directly inoculating the human subject with the matter called grease, furnished by the diseased heels of the horse, is "indisputably safe and efficacious." This phrase is not marked with inverted commas, denoting it to be a literal quotation; it was employed to describe the confidence which is obviously implied in the passage cited in my last paper on the subject. Has Mr. Ring any right to complain that he is misrepresented in being charged with having thought the practice "indisputably safe and efficacious," after affirming "that the disease called the genuine cow-pox proceeds from the horse, is as clearly proved as any other fact in medical science, a similar disease, endowed with similar prophylactic virtue, having been repeatedly produced in the human subject, without the intervention of the cow. This, among other instances, has been done by Dr. Sacco, of Milan, who was before sceptical on the subject, in the presence of several medical men assembled for that purpose, one of whom was a friend of Dr. De Carro. Matter of this kind has been transmitted to Dr. De Carro, and to Dr. Friese of Silesia, and has also succeeded in their hands. They now inoculate indiscriminately with equine or vaccine matter, and with equal effect." How can Mr. Ring suppose an expression to be "unguarded," which appears to be fully warranted by the most unquestionable testimony? Either Mr. Ring's own quotation, and his own opinion of it, has not the authority it would seem to possess, or it abundantly justifies the inference, that he esteems the practice of directly inoculating the human subject with equine matter as "indisputably safe and efficacious."

Several instances cited by Mr. Ring, in the last Number of your Journal, are highly corroborative of his opinion, that the vaccine pock has its origin in a diseased secretion of the horse. But on the authority of this evidence nothing precise seems to be ascertained as to the particular *mode* of grease, or the *period* when it exerts its specific infection. It is too vague, therefore, to ascribe the origin of of a disease to what is generally called *grease*, when it probably derives its existence from a *form* or *stage* of the affection, which that general term by no means points out. If what is denominated *grease* has no effect when inserted into the cow's teat in one instance, and produces a specific influence in another, it evinces an essential difference in the circumstances of the experiment, and proves that the investigation is yet rather in its infancy, than become "hackneyed and threadbare," as Mr. Ring insists on. If the subject is "hackneyed and threadbare," the labour which has so exhausted it, has been for the most part fruitless. Either the research is extremely difficult, or it has not been conducted in a manner calculated to insure success.

Casual instances of diseases, apparently arising from the horse, resembling the cow-pock affection, deserve all the credit due to accidental information; but unless the hint is taken up, and carried to demonstrative proof by a regular series of unequivocal experiments, it has no title to be considered as a philosophical axiom, nor to be regarded with the boastful confidence in its unquestionable clearness, discovered by Mr. Ring. It should not be forgotten, that various states of herpetic affection, occasionally occurring on the hands of the milkers, who may by chance also be employed in the care of horses, may possibly impart to the cow a disease, which a mere coincidence of circumstances may have attributed to the horse. If the *grease*, or rather "scratchy heel," as has been more diagnostically remarked, were either uniformly, or in a large majority of instances, to induce the disease called vaccine pock, all doubt would be at an end respecting its origin. It will therefore be my future endeavour to present the public, and particularly Mr. Ring, with such evidence on this subject as shall either confirm his authorities for the opinion he entertains, or at least "shake" if not refute them, however zealously or voluminously he may have written in their defence.

The farmer and carter, who attended my experiments, though treated with sarcastic levity by Mr. Ring, are, on the question relating to a greasy heeled horse, very admissible authorities; more so than Mr. Ring himself can pretend to be, who must be allowed to be more conversant with declaiming on the subject, than with practically examining its merits, either at its fountain head, or "fountain heel," as he would wittily phrase it.

I have lately been assured by two persons, extensively concerned concerned in the management of dairies, that the cow-pock disease, when it occurs, generally happens in the course of milking after the first cast; that it approaches by a soreness of the teats, discoverable by an impatience in the animal to be milked; after a few days, pustular eruptions appear, containing a thin transparent fluid; soon after these vesicles break, and leave the open sore that is so troublesome to the cow in milking. These persons (who are very intelligent in their dairy occupations) naturally ascribe the disease to the violence done the teats in first handling them in the act of milking, as they do not observe a similar occurrence in the older cows, which have been accustomed to that operation. If it be a fact that cows are more liable to the disease on being first milked, it may probably be in some measure owing to the process of lactescence disposing the breast and nipples of the animal to inflammatory affection. This is observable in the human subject, and more particularly after the first child. It is well known that the nipples, in these circumstances, are often rendered so sore by the action of suckling, as to become an insuperable obstacle to pursuing that humane practice.

One of the persons alluded to, assured me that the milking of the cows, which in different years had severely suffered from cow-pock ulceration, was exclusively confined to female servants, who in no respect were liable to come in contact with the infectious grease of the horses heel. The same person also informed me, that the hands of her female servants had been several times more or less inflamed and ulcerated, by milking the cows, whose teats had been excoriated in the manner stated.

The disease was always considered as infectious, and was actually transferred to other cows, by the hands of the person who had been employed in milking the distempered cow.

If the disease should appear thus to arise spontaneously, it would at least prove that it is not exclusively owing to the grease of the horses heel, and would farther warrant a suspicion that any kind of acrimony, whether induced by the specific properties of any particular disease, or more casually generated by accidental inflammation, might be capable of producing cow-pock affection. Mr. Ring quotes Dr. Jenner's opinion, that an inflammatory action in any part of the horse, may furnish the virus of cow-pock disease. This opinion is founded on the fact of "an extensive inflammation of the erysipelatous kind having taken taken place on the upper part of the thigh of a colt, belonging to Mr. Millet, of Rockhampton. This inflammation was followed by sores, which were dressed by the same persons who were employed in milking. Soon after, all the cows and milkers in the dairy were affected with a disease, which appeared to be the genuine cow-pox." On parity of reason, may not an inflammation, originating either on the teats, or any other part of the cow, afford an acrimony capable of similar effects? The occasional failure of cow-pock inoculation, in its preventive efficacy against small-pox, would appear to be owing to at least the semblance of that disease, being produced by acrimony arising in different circumstances of vascular action. It will therefore be of the utmost importance, to ascertain the true source of this disease, with infinitely more precision than has been yet done, by either the meritorious Dr. Jenner himself, or by his elaborate expositor, Mr. Ring.

Mr. Ring may deem what has been hitherto done in this inquiry, as sufficiently conclusive to exempt himself from the trouble of farther research on the subject; but his confidence will not satisfy the desires of those, who may be less persuaded than he is, of the adequacy of that testimony. Mr. Ring may leave my doubts unsatisfied by his own exertions in the investigation, but that avowal of indifference should not restrain an assiduous endeavour, to elucidate what appears at present to be enveloped in impenetrable obscurity. I therefore again pledge myself to enter on a course of experiments, that must lead to a result, not unworthy of even Mr. Ring's attention, however decisively he may have been convinced, by the weight of authority which he has adduced, and which he conceives renders all *direct* investigation from himself superfluous, and "interfering too much with other duties, which he deems still more important."

I am, &c.

ROBERT KINGLAKE.

*Taunton, August* 12, 1806.

